# Pathogenic Mechanisms and Host Interactions in *Staphylococcus epidermidis* Device-Related Infection

**DOI:** 10.3389/fmicb.2017.01401

**Published:** 2017-08-02

**Authors:** Marina Sabaté Brescó, Llinos G. Harris, Keith Thompson, Barbara Stanic, Mario Morgenstern, Liam O'Mahony, R. Geoff Richards, T. Fintan Moriarty

**Affiliations:** ^1^Musculoskeletal Infection, AO Research Institute Davos Davos, Switzerland; ^2^Molecular Immunology, Swiss Institute of Allergy and Asthma Research, University of Zurich Davos, Switzerland; ^3^Microbiology and Infectious Diseases, Institute of Life Science, Swansea University Medical School Swansea, United Kingdom; ^4^Department of Orthopedic and Trauma Surgery, University Hospital Basel Basel, Switzerland

**Keywords:** *Staphylococcus epidermidis*, coagulase-negative staphylococci, commensal bacteria, device-related infection, bone infection, biofilm, immune responses

## Abstract

*Staphylococcus epidermidis* is a permanent member of the normal human microbiota, commonly found on skin and mucous membranes. By adhering to tissue surface moieties of the host via specific adhesins, *S. epidermidis* is capable of establishing a lifelong commensal relationship with humans that begins early in life. In its role as a commensal organism, *S. epidermidis* is thought to provide benefits to human host, including out-competing more virulent pathogens. However, largely due to its capacity to form biofilm on implanted foreign bodies, *S. epidermidis* has emerged as an important opportunistic pathogen in patients receiving medical devices. *S. epidermidis* causes approximately 20% of all orthopedic device-related infections (ODRIs), increasing up to 50% in late-developing infections. Despite this prevalence, it remains underrepresented in the scientific literature, in particular lagging behind the study of the *S. aureus*. This review aims to provide an overview of the interactions of *S. epidermidis* with the human host, both as a commensal and as a pathogen. The mechanisms retained by *S. epidermidis* that enable colonization of human skin as well as invasive infection, will be described, with a particular focus upon biofilm formation. The host immune responses to these infections are also described, including how *S. epidermidis* seems to trigger low levels of pro-inflammatory cytokines and high levels of interleukin-10, which may contribute to the sub-acute and persistent nature often associated with these infections. The adaptive immune response to *S. epidermidis* remains poorly described, and represents an area which may provide significant new discoveries in the coming years.

## Introduction

*Staphylococcus epidermidis* is a permanent member of the normal human microbiota, commonly found on skin, and mucous membranes. By adhering to tissue surface moieties of the host via specific adhesins, *S. epidermidis* is capable of establishing a lifelong commensal relationship with humans that begins early in life. Although commensal *S. epidermidis* isolates display high rates of resistance to antibiotics of clinical relevance (Morgenstern et al., 2016a), their default status as commensal bacteria renders this phenomenon largely irrelevant for the healthy human host. However, with the advent of implanted medical devices such as prosthetic joints and fracture fixation devices, *S. epidermidis* has emerged as an important opportunistic pathogen (Otto, 2009; Widerstrom, 2016). In fact, the implanted medical device may actually facilitate infection since any *S. epidermidis* inadvertently introduced into the surgical site are capable of rapidly adhering to, and accumulating upon, the surface of the device. This surface-associated bacterial growth is known as biofilm formation and appears to be the key factor enabling invasive, device-related infection (DRI) for an otherwise largely non-pathogenic microorganism. The ubiquitous presence of *S. epidermidis* on human skin has enabled *S. epidermidis* infection to emerge as a significant complication when using medical devices (Rogers et al., 2009; Montanaro et al., 2011; Hogan et al., 2015). With the increasing use of such devices, coupled with high antibiotic resistance rates, *S. epidermidis* DRI will likely remain a clinical problem for generations to come.

This review describes host interactions with *S. epidermidis* under both healthy commensal conditions, and under conditions of an invasive DRI. This includes describing how this microorganism has adapted to life on human skin, including biofilm formation, and how the same adaptations have enabled invasive DRI. Particular attention will be paid to the impact of *S. epidermidis* in orthopedic device-related infection (ODRI) since these infections are amongst the most burdensome and expensive to treat (Darouiche, 2004). Finally, since the impact of ODRI on bone tissue is a critical feature of these infections, interactions between *S. epidermidis* and bone will also be described.

## *S. epidermidis* as a member of commensal human microbiota

Under healthy conditions, the skin commensal microbiota is believed to be beneficial to humans through aiding in nutrition, outcompeting pathogens and educating the immune system (Brown and Clarke, 2017). Humans are believed to first encounter *S. epidermidis in utero*, as evidenced by their presence in amniotic fluid (Collado et al., 2016). The first feces (meconium) has also been shown to harbor a predominance of *S. epidermidis* (Jimenez et al., 2008) and the skin of the newborn will be colonized by *S. epidermidis* within a few days (Dominguez-Bello et al., 2010). Thereafter, *S. epidermidis* becomes part of the “normal” resident human skin microbiota, being predominant in moist sites such as nares or fossae, but also present in sebaceous areas such as the facial skin (Grice et al., 2009) and mucosal tissues such as the gastrointestinal and the lower reproductive tracts (Sharon et al., 2013; Majchrzak et al., 2016).

In order to persist on human skin, *S. epidermidis* has evolved diverse mechanisms to sense and overcome the physical and chemical features of host antimicrobial defense. Such mechanisms include surface adhesins enabling attachment to the host (Coates et al., 2014), systems to sense host antimicrobial peptides (AMPs) and communication molecules (e.g., hormones) (Li et al., 2007; N'Diaye et al., 2016), mechanisms against AMPs (Joo and Otto, 2015) (e.g., *S. epidermidis* derived protease SepA is induced by and directed against the human AMP dermicidin; Lai et al., 2007), and survival strategies against desiccation and osmotic stress (Hirai, 1991; Amin et al., 1995).

*S. epidermidis* has also been shown to influence host colonization by other species, as shown for *Staphylococcus aureus* (Iwase et al., 2010; Park et al., 2011). Negative correlations between these two species have been reported in humans, insinuating an antagonism between at least some strains (Frank et al., 2010; Sullivan et al., 2016). This effect is at least partially due to the secretion of factors that impact on the viability or colonization capacity of other microorganisms (Christensen et al., 2016; Janek et al., 2016). Phenol soluble modulins (PSMs) are a family of multifunctional amphipathic, alpha-helical peptides that are produced by *S. epidermidis* isolates (Otto, 2014). They are believed to act upon host cells, are important for biofilm maturation (Wang et al., 2011) and could play a role in the competition between microorganisms on human skin. In particular, PSM-γ and PSM-δ produced by *S. epidermidis* have been shown to selectively reduce survival of *Streptococcus pyogenes* on mouse skin, but did not affect *S. epidermidis* itself (Cogen et al., 2010a,b). Both PSM-γ and PSM-δ cause membrane leakage in target bacteria (*S. aureus* and *S. pyogens*) (Cogen et al., 2010b), which indicates that they function like host-derived AMPs, with whom they share structural similarities. Host-derived AMPs and *S. epidermidis* PSMs have even been shown to act synergistically against bacterial pathogens (Cogen et al., 2010a). In contrast, the closely related δ-toxin of *S. aureus* only seems to possess a very limited antimicrobial activity (Dhople and Nagaraj, 1993, 2005) suggesting that the cooperative effect with host AMPs is not a widespread phenomenon. In addition, many strains of *S. epidermidis* also produce bacteriocins, which are antimicrobial peptides that act against other species or strains (often closely related to the producing bacteria). Gram-positive bacteria usually produce two types of bacteriocins: lanthionine-containing antibacterial peptides (lantibiotics) and class-II bacteriocins (Bastos et al., 2009; Hassan et al., 2012). For *S. epidermidis*, examples include the lantibiotics epidermin (Allgaier et al., 1986), Pep5, epilancin K7 (van de Kamp et al., 1995), and epilancin 15X (Ekkelenkamp et al., 2005), with further examples recently described (Sandiford and Upton, 2012; Bennallack et al., 2014; Janek et al., 2016). Another mechanism employed by *S. epidermidis* to compete with other skin microorganisms involves the degradation of biofilms from other bacterial species. The serine protease Esp is able to mediate *S. aureus* biofilm degradation by targeting several proteins involved in biofilm assembly (Iwase et al., 2010; Sugimoto et al., 2013). It has been observed that the presence of Esp-secreting *S. epidermidis* in the nose correlates with the absence of *S. aureus* in healthy human volunteers (Iwase et al., 2010). This activity has been supported experimentally with the finding that the intranasal application of an Esp-secreting strain was able to decrease *S. aureus* colonization in mice and humans (Iwase et al., 2010; Park et al., 2011). Finally, metabolic products may also serve to counteract other microorganisms. *S. epidermidis* has been shown to ferment glycerol into short chain fatty acids, which have displayed inhibitory activity against *Propionibacterium acnes* (implicated in acne vulgaris) *in vitro* and in mice (Wang et al., 2014).

## *S. epidermidis* as a pathogen

In contrast to its standard role as a commensal microorganism, *S. epidermidis* and other coagulase negative Staphylococci (CoNS) have been found to cause invasive infections in selected groups of patients. These higher risk groups include preterm neonates, immunocompromised individuals and patients with indwelling medical devices (Darouiche, 2004; Bjorkqvist et al., 2010; Dong and Speer, 2014). Unlike *S. aureus*, which typically produces numerous extracellular enzymes and toxins that enable invasive infections in otherwise healthy hosts, *S. epidermidis* seems to retain a limited number of virulence factors (Gill et al., 2005) and normally is unable to cause invasive infection in healthy hosts (Heilmann and Gotz, 2013).

### *S. epidermidis* as a pathogen of the musculoskeletal system

*S. epidermidis* is second only to *S. aureus* as the most prevalent species encountered in ODRIs (Trampuz and Zimmerli, 2005, 2006). *S. epidermidis* causes approximately 20–30% of ODRIs (Trampuz and Zimmerli, 2006; Montanaro et al., 2011; Moriarty et al., 2016) and the prevalence may even increase to 50% in late-developing infections (Schafer et al., 2008). These late-developing infections may be linked to the sub-acute nature of *S. epidermidis* infections, which may present many months after surgery with subtle signs of infection. This differs from the acute and often obvious nature of *S. aureus* infections and may be partially explained by the lack of virulence factors retained by *S. epidermidis* in comparison with *S. aureus* (Melzer et al., 2003; Zimmerli et al., 2004; Shurland et al., 2007).

The diagnosis of ODRI is based on the combination of clinical presentation, biopsy culture, histological analysis and clinical diagnostic criteria, such as high C-reactive protein (Metsemakers et al., 2016). Diagnosis may be particularly challenging for sub-acute infections due to the lack of obvious clinical signs of infection. Therefore, microbiological culture results are often the most critical diagnostic criteria. Since the microbes grow in biofilms on the foreign material and in necrotic bone tissue, cultivation and identification of the disease-causing pathogens may require the culture of several intraoperative tissue samples and removal of the implant for appropriate sampling (Costerton et al., 2011; Xu et al., 2017). To increase the yield of positive cultures, it is advised to terminate antibiotic therapy before sampling, acquire at least three tissue biopsies, and to perform sonication of removed hardware to remove biofilm-associated bacteria from the surface (Trampuz and Zimmerli, 2006; Trampuz et al., 2007; Puig-Verdie et al., 2013; Yano et al., 2014; Dapunt et al., 2015; Metsemakers et al., 2016). In suspected *S. epidermidis* infections, where the pathogen is also a skin commensal that could contaminate the biopsy if aseptic techniques are not followed, the same indistinguishable microorganism must be cultured from at least two separate biopsies in order to differentiate a relevant infection from skin contamination. In contrast, in virulent species such as *S. aureus* or *Escherichia coli*, a single positive biopsy may be sufficient to determine the presence of an infection (Patzakis and Zalavras, 2005; Osmon et al., 2013).

The treatment of *S. epidermidis* ODRI will depend on patient-specific factors, but will possibly require implant removal and a minimum of 6 weeks antibiotic therapy (Trampuz and Zimmerli, 2005, 2006; Moriarty et al., 2016). Despite such prolonged and comprehensive therapy, infection recurs in approximately one third of the cases and up to one fifth of cases cannot achieve a cure with restoration of limb function (Salgado et al., 2007; Teterycz et al., 2010; Morgenstern et al., 2016b,c). Morgenstern et al. investigated the clinical course and outcome of staphylococcal ODRIs in elderly patients and could show that *S. epidermidis* was associated with prolonged infections and was associated with lower cure rates (75%) than *S. aureus* (84%), although *S. aureus* related infections were associated with a five-fold higher mortality rate (Morgenstern et al., 2016b). This data therefore supports clinical beliefs that *S. epidermidis* is an agent of sub-acute infection with significantly worse treatment outcomes, although those infections may be less life-threatening than *S. aureus* infections.

### *S. epidermidis* virulence factors

#### Adhesion to host proteins

**Figure 1:**
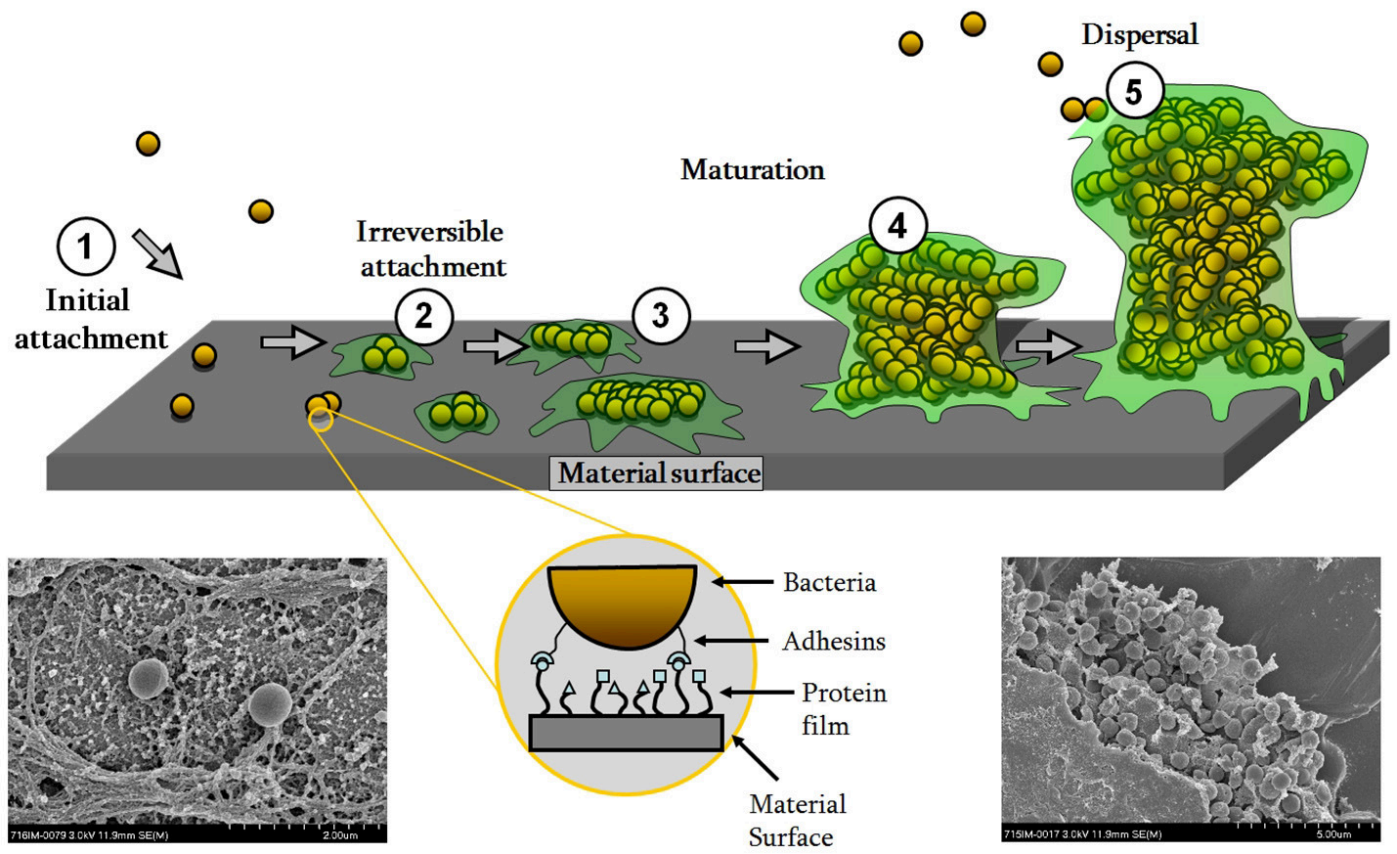
Biofilm formation scheme with scanning electron micrographs of *S. epidermidis* single cells (lower left) or in biofilm community surrounded by EPS (lower right) on a titanium surface. Image adapted with permission from Moriarty et al. (2011).

**Figure 2:**
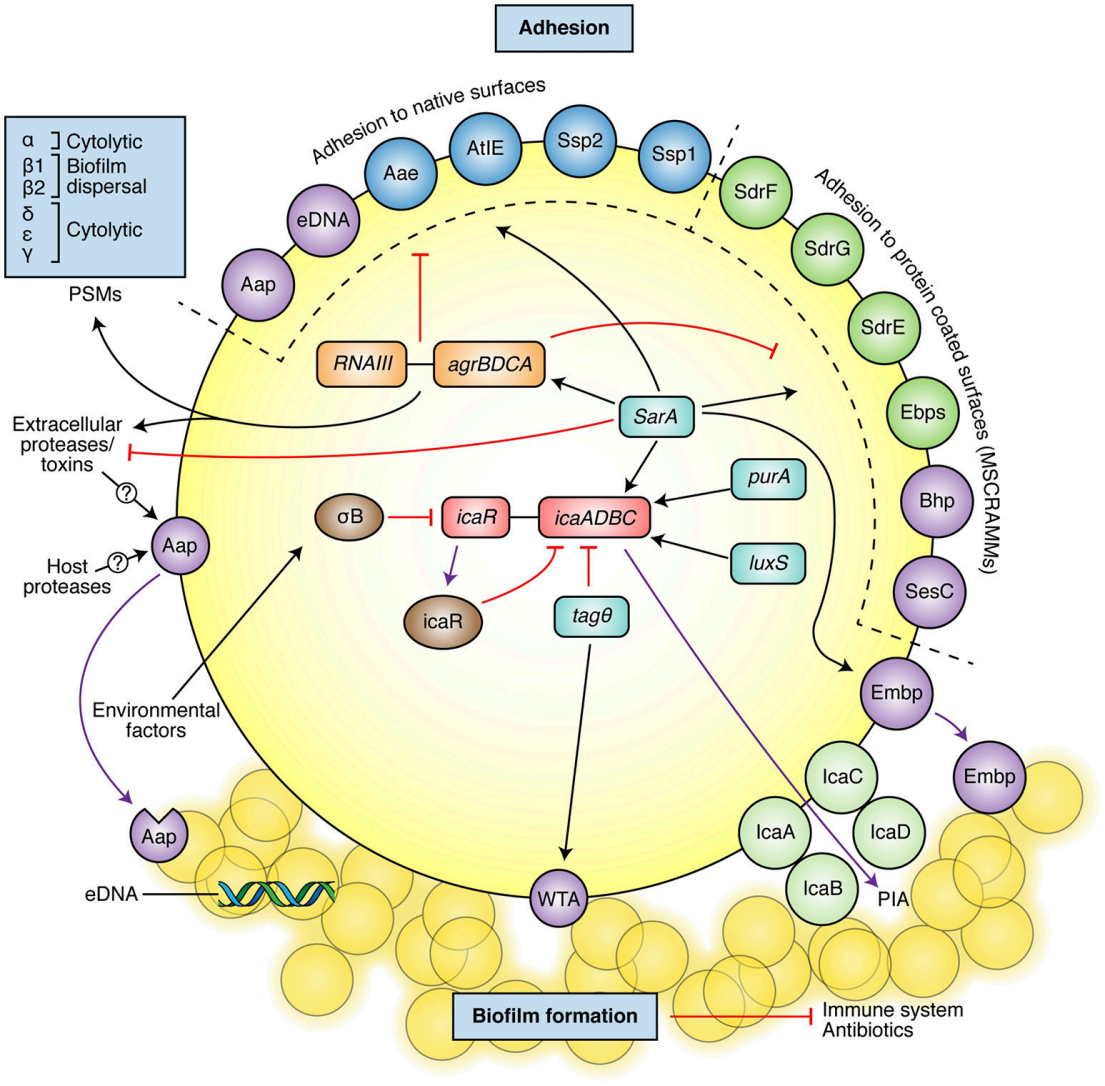
Scheme of the main *S. epidermidis* pathogenic mechanisms, which include adhesion molecules and biofilm formation. The most well described adhesins involved in adhesion to native surfaces or protein-coated surfaces are shown in the upper part (molecules also involved in biofilm formation shown in purple). The main described biofilm components are shown at the bottom of the figure (PIA, cleaved Aap, eDNA, WTA, and Empb). The figure also presents some of the most important regulators of biofilm and adhesion molecules (black arrows: activation/positive signaling, red lines: inhibition/negative signaling). See text for further details.

As a commensal microorganism, *S. epidermidis* retains the ability to specifically adhere to host proteins in the skin. In a surgical wound, the bacterium utilizes these adhesion mechanisms in order to adhere to the deeper tissues and to the implanted device, or more specifically, the conditioning layer of host proteins deposited upon the device. Initial adhesion of bacteria to implant surfaces is mediated by non-specific interactions such as hydrophobic interactions (Gristina, 1987), and then as shown schematically in Figure 1, by specific adhesins such as autolysin (AtlE) (Heilmann et al., 1997), extracellular DNA (eDNA) (Qin et al., 2007; Izano et al., 2008), and staphylococcal surface protein 1 and 2 (SSP-1, SSP-2) (Veenstra et al., 1996). AtlE, SSP-1, and SSP-2 have been primarily associated with adhesion to native surfaces (Veenstra et al., 1996; Heilmann et al., 1997), whilst eDNA is generated in *S. epidermidis* through an AtlE-mediated lysis of a subpopulation of the bacteria, promoting biofilm formation within the remaining population (Qin et al., 2007). In the context of medical devices, the surface of the device becomes coated with host-derived plasma proteins, extracellular matrix (ECM) proteins and coagulation products (platelets and thrombin) immediately following implantation (Baier et al., 1984). Cell-wall-anchored (CWA) proteins/adhesins, such as the microbial surface components recognizing adhesive matrix molecules (MSCRAMMs) (Foster and Hook, 1998) bind bacteria like *S. epidermidis* directly to these molecules (Figure 2). In *S. epidermidis*, adhesins for fibrinogen [as serine-aspartate repeat protein G (SdrG/Fbe) (Hartford et al., 2001; Brennan et al., 2009)], fibronectin [extracellular matrix-binding protein (Embp) (Arciola et al., 2003)], collagen [SdrF/GehD (Bowden et al., 2002; Arrecubieta et al., 2007)], vitronectin [AtlE or autolysin/adhesin (Aae) (Heilmann et al., 2003)] and elastin [elastin-binding protein (EbpS)] have all been identified. Peptidoglycan-bound wall teichoic acids (WTA) are an essential part of the *S. epidermidis* cell wall and also play an important role in bacterial adhesion. WTA enhances the initial adhesion of *S. epidermidis* to medical devices by binding to adsorbed fibronectin (Hussain et al., 2001) and fibrin clots (Chugh et al., 1990).

#### Biofilm formation

The ability to adhere to a surface represents the first step in biofilm formation, commonly believed to be the most important virulence factor possessed by *S. epidermidis* (Figure 1). Biofilm development facilitates resistance against host defense mechanisms (Myrvik et al., 1989; Kristian et al., 2008; Cerca et al., 2011; Schommer et al., 2011) and confers antibiotic resistance (Cerca et al., 2006; Mack et al., 2006). Biofilm formation also complicates medical and surgical treatment protocols because implant removal is often required to remove the biofilm.

Biofilms are defined as complex communities of adherent bacteria encased in a matrix of self-produced extracellular polymeric substances (EPS) (Costerton et al., 1995) (Figure 1). The accumulation and maturation of the *S. epidermidis* biofilm occurs via a number of mechanisms. Polysaccharide intercellular adhesin [PIA, or poly-N-acetyl-glucosamine (PNAG)], synthesized by *icaADBC* encoded proteins (Heilmann et al., 1996; Mack et al., 1996a) is responsible for biofilm formation in the majority of *S. epidermidis* isolates (Mack et al., 1996b) and was believed to be the most common molecule associated with biofilm formation (Heilmann et al., 1996; Mack et al., 1996a; Figure 2). This was endorsed by the observation that the *ica* operon was absent in most commensal *S. epidermidis* strains (Zhang et al., 2003; Chokr et al., 2007). However, not all *S. epidermidis* have the *icaADBC* genes (Heilmann et al., 1996; Harris et al., 2016) and these isolates mediate biofilm formation by proteinaceous factors, such as the accumulation associated protein (Aap) (Rohde et al., 2005) that contributes to biofilm formation upon cleavage by extracellular or host proteases (Figure 2). The *aap* gene has been observed in both pathogenic and commensal isolates, more frequently than the *ica* operon (Gill et al., 2005; Los et al., 2010; Harris et al., 2016). Other PIA-independent mechanisms include biofilm associated homolog protein (Bhp) (Bowden et al., 2005; Tormo et al., 2005), Embp (Williams et al., 2002; Christner et al., 2010), and *S. epidermidis* surface protein (Ses)C (Shahrooei et al., 2009), and SesE (Harris et al., 2016). Interestingly, Rohde et al. suggested that PIA-dependent biofilms are more robust than those formed by proteinaceous factors (Rohde et al., 2007), and another study found they result in a different morphotype or biofilm substructure (Harris et al., 2016). WTA have also been linked with *S. epidermidis* biofilm formation. *TagO* encodes the first enzymatic step in WTA biosynthesis and a *tagO* mutant has been shown to have a biofilm negative phenotype. This is partly attributed to an increase in cell surface hydrophobicity, impairing its initial adhesion to the surface, and a decreased production of PIA by activating the *icaADBC* repressor, *icaR* (Holland et al., 2011).

Both CWA proteins and biofilm formation mechanisms are regulated by several global regulators, such as the accessory gene regulator (*agr*), staphylococcal accessory homologous *sar* genes, sigma factor B (σ^B^), and *luxS* (Vuong et al., 2003; Knobloch et al., 2004; Xu et al., 2006; Christner et al., 2012). Further information on regulation of biofilm in *S. epidermidis* can be obtained in other review articles (Kong et al., 2006; Mack et al., 2007; Le and Otto, 2015; Paharik and Horswill, 2016).

As already mentioned, biofilms play a role in immune evasion, primarily by providing a barrier to immune cells. PIA may contribute to innate immune system evasion by promoting generation of complement C5a fragment (Satorius et al., 2013; Al-Ishaq et al., 2015), inhibiting phagocytes and neutrophil killing (Vuong et al., 2004b,c), and reducing the activity of AMPs (Vuong et al., 2004b; Otto, 2006). Recently, other studies have reported slightly opposite findings, with PIA-producing bacteria inducing greater inflammatory responses and enhanced phagocytosis (Spiliopoulou et al., 2012; Ferreirinha et al., 2016), although Spiliopoulou et al. did observe reduced killing in PIA-producing strains as discussed elsewhere recently (Nguyen et al., 2017). *S. epidermidis* also produces a second exopolymer, the poly-γ-glutamic acid (PGA), although at comparatively lower levels. Synthesized by the gene products of the *cap* locus, PGA is important in mediating *S. epidermidis* resistance to neutrophil phagocytosis and AMPs, and promoting growth at high salt concentrations (PGA is induced under such conditions) (Kocianova et al., 2005).

It has yet to be elucidated if WTA has a direct role in *S. epidermidis* immune system evasion. However, like *S. aureus, S. epidermidis* contains the genes for D-alanylation of WTA, a modification known to protect the bacteria from the activity of AMPs (Peschel et al., 1999).

#### Antibiotic resistance

Although the majority of *S. epidermidis* strains remain susceptible to the newer antibiotics such as daptomycin, tigecycline, linezolid and dalbavancin (Hellmark et al., 2009; Pinheiro et al., 2016), high endemic antimicrobial resistance within this species represents a significant challenge in the treatment of *S. epidermidis* infections, especially DRI (Diekema et al., 2001). Methicillin resistance in *S. epidermidis* (MRSE) is an important characteristic of infecting isolates as it is often associated with additional antibiotic resistance mechanisms. Resistance to other antibiotics, such as erythromycin (encoded by *erm* genes), ciprofloxacin, clindamycin, aminoglycosides (encoded in *aacA/aphD* gene) or trimethoprim-sulfamethoxazole, are also often observed, especially in MRSE (Cherifi et al., 2013). Methicillin resistance is encoded by *mecA*, an alternative penicillin binding protein with decreased affinity to β-lactam based antibiotics such as penicillin, methicillin and oxacillin (Chambers et al., 1985). It is carried on the mobile genetic element, staphylococcal cassette chromosome *mec* (SCC*mec*), of which several types have been identified for *S. epidermidis* (Miragaia et al., 2005). MRSE have been found to be common in infection-causing isolates (70–87% of all *S. epidermidis* isolates) (Cherifi et al., 2013; Farina et al., 2016; Morgenstern et al., 2016c; Salgueiro et al., 2017), and even higher (90%) in specific patient cohorts (Morgenstern et al., 2016b). MRSE prevalence in healthy individuals is low (3–18% of *S. epidermidis* commensal isolates) (Rolo et al., 2012; Cherifi et al., 2013; Farina et al., 2016), although prevalence is increased for individuals exposed to the healthcare system, as observed in hospitalized patients or in healthcare workers (Rohde et al., 2004; Morgenstern et al., 2016a; Widerstrom et al., 2016). The specific causes of the increased prevalence of resistant isolates in the hospital environment is unknown, although is likely associated with high antibiotic exposure and direct or indirect interpersonal transmission.

It remains unclear whether infection with resistant organisms results in a worse clinical outcome in comparison with susceptible counterparts. In a recent study of patients with *S. epidermidis* ODRIs, methicillin resistance status did not influence the clinical course and outcome of treatment (Morgenstern et al., 2016c), although further studies are required to confirm this finding. In any case, clear therapeutic guidelines are available for the treatment of both MRSE and MSSE, with a high likelihood of treatment success in both cases when guidelines are followed closely.

#### Phenol soluble modulins

Until relatively recently it was thought that *S. epidermidis* did not produce toxins. However, the identification and characterization of the PSMs have now changed that concept (Mehlin et al., 1999). The PSMs are a family of genome-encoded peptides, and like the CWA proteins/adhesins, are under the strict regulation of the *agr* quorum sensing system (Figure 2; Mehlin et al., 1999; Vuong et al., 2004a; Yao et al., 2005). In *S. epidermidis*, the PSM family consists of PSM-α, PSM-β1, PSM-β2, PSM-δ, PSM-ε, and PSM-γ/δ-toxin (Mehlin et al., 1999; Vuong et al., 2004a; Yao et al., 2005). PSMβ peptides are the primary PSMs produced by *S. epidermidis*, are expressed at high levels during biofilm formation, and have been shown to have a role in the structuring and dispersal of the biofilm (Yao et al., 2005; Wang et al., 2011). They are specifically associated with the formation of channels observed between the biofilm layers, which are considered important for nutrient uptake (Wang et al., 2011). *S. epidermidis*-derived PSMδ is strongly cytolytic against neutrophils, similar to *S. aureus*. However, *S. epidermidis* culture filtrates were observed to have a very low cytolytic potential *in vitro* (Cheung et al., 2010). As growing conditions are likely to have an influence on PSM production, the role of *S. epidermidis* PSMδ *in vivo* needs to be further addressed.

Finally, certain *S. epidermidis* strains have been shown to produce PSM-mec, a PSM encoded in the mobile genetic element SCC*mec*, in contrast to the other PSMs that are chromosomal encoded (Qin et al., 2016). PSM-mec has cytolytic potential against neutrophils *in vitro* and its presence has been associated with decreased bacterial clearance and higher mortality rates in a murine model of sepsis (Qin et al., 2017).

#### Other pathogenic mechanisms

Small colony variants (SCVs), a colony phenotype characterized by small size, slow growth and downregulation of virulence genes, are recognized as a pathogenic mechanism for several bacterial species, including *S. epidermidis*, and are often associated with chronic infections (Johns et al., 2015). SCVs seem to be less susceptible to antibiotics and to the immune system, potentially by being able to survive intracellularly and inducing a more anti-inflammatory environment due to increased secretion of IL-10 (Magrys et al., 2015). The topic has been extensively reviewed recently (Kahl et al., 2016).

Finally, internalization and intracellular persistence in non-professional phagocytes (e.g., osteoblasts) is a described evasion mechanism for *S. aureus* (Mempel et al., 2002; Hamza and Li, 2014). A few internalization mechanisms have been described for *S. epidermidis*, involving AtlE (Hirschhausen et al., 2010) and SdrG (Claro et al., 2015). This represents a potentially new pathogenic mechanism for *S. epidermidis* and a location where bacteria could survive to cause persistent/relapsing infections; however its relevance *in vivo* has not yet been proven.

## Host interaction with *S. epidermidis*

The interaction between *S. epidermidis* as a commensal with the host immune system is thought to play a role in the development of immunological tolerance. That is, to induce immune responses in the host which control aberrant inflammatory responses to non-pathogenic molecules such as those found in food but also in commensal bacteria. This question was assessed in recent murine studies with the topical application of *S. epidermidis* (Naik et al., 2015; Scharschmidt et al., 2015) (*S. epidermidis* is typically not a major representative of the normal mouse skin microbiota; Tavakkol et al., 2010). Scharschmidt et al. reported that the application of *S. epidermidis* to the skin within the first weeks of life established antigen-specific tolerance to the bacteria, by generating CD4+ regulatory T (Treg) cells, which homed into neonatal skin (Scharschmidt et al., 2015). Mice that were not colonized during the neonatal period presented with higher inflammation and neutrophil recruitment compared to colonized mice, when challenged with the same strain of *S. epidermidis* in a skin-abrasion model. The use of the sphingosine-1-phosphate receptor antagonist FTY720 during neonatal period, which blocked the egression of Tregs into skin, suppressed the tolerogenic effect indicating that there may exist a critical period when Treg mediated tolerance can be acquired (Scharschmidt et al., 2015). On the other hand, Naik et al. showed that *S. epidermidis* application induced cutaneous interferon (IFN)-γ and interleukin (IL)-17A producing T cells (Naik et al., 2015). In this case, IL-17A+CD8+ T cells were shown to home to the mouse epidermis specifically after *S. epidermidis* application, but not with other tested species. This was mediated through the action of a skin-resident dendritic cell subset and was not associated with the induction of inflammation (Naik et al., 2015). More importantly, when an epicutaneous infection model with *Candida albicans* was used, the application of the fungus in mice pretreated with topical *S. epidermidis* resulted in decreased *C. albicans* CFU counts compared to not pretreated ones. The effect was lost when either anti-CD8 or anti-IL-17A antibodies were co-administered, which highlights the relevance of the adaptive immune responses generated. Altogether, the study suggested that resident bacteria in the skin (*S. epidermidis*) can modulate the immune system, generating adaptive immune responses which in turn may help in promoting protective innate immune responses and controlling inflammation. The effect seemed to be tissue-specific, since *S. epidermidis* failed to induce IL-17A-producing cells when administered in the lung or gut. In two other studies, *S. epidermidis* lipoteichoic acid (LTA) has been shown to decrease skin inflammation (Lai et al., 2009), for example by inducing regulatory microRNAs in a *Pseudomonas aeruginosa* skin infection model (Xia et al., 2016). However, the true nature of these observations needs to be clarified, as LTA purity even from commercial preparations has been questioned (Nguyen et al., 2017).

Overall, these experimental data reveal the capacity of “commensal” *S. epidermidis* to specifically shape cutaneous immunity (innate and adaptive responses) and consequently decrease infection burden in the host. The capacity of *S. epidermidis* to induce similar effects in humans remains to be proven. Nevertheless, this idea can be somewhat supported by *in vitro* findings, whereby human monocytes, monocyte-derived dendritic cells (moDC) and T lymphocytes stimulated with *S. epidermidis* displayed an anti-inflammatory profile, with high production of IL-10 (Laborel-Preneron et al., 2015). Further *in vivo* and human microbiome studies may provide a deeper understanding of the complex nature of this microorganism-host interaction.

### Innate immune response during infection

#### Recognition

**Figure 3:**
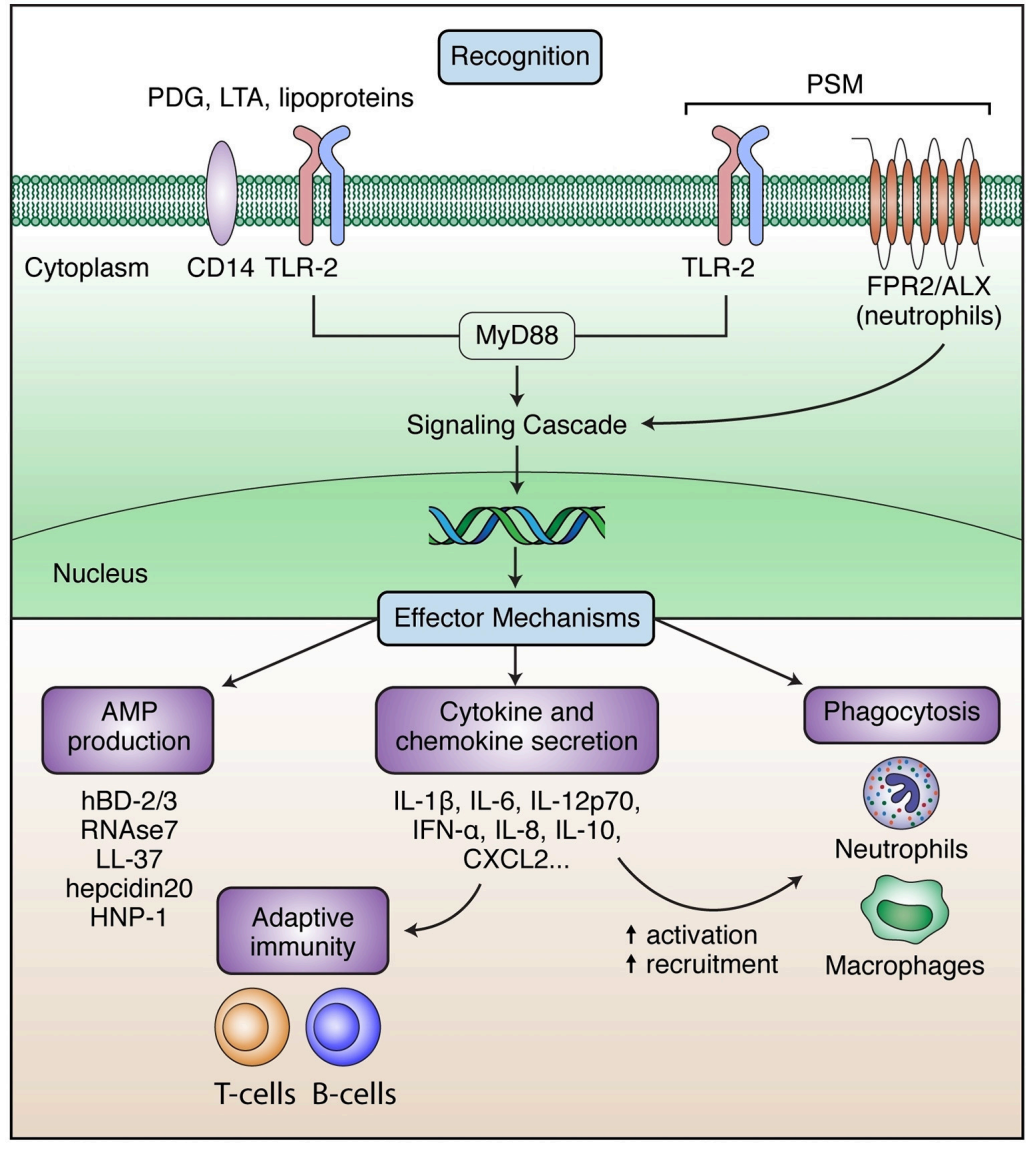
Summary of *S. epidermidis* recognition and subsequent effector mechanisms. Recognition of *S. epidermidis* or its secreted proteins can occur via TLR-2 (in red), which forms heterodimers with TLR-1 and TLR-6 and can also associate with other non-TLR molecules (unspecified partner colored in blue). Other receptors recognizing *S. epidermidis* include CD14 and FPR2/ALX. Upon recognition, downstream signaling and effector mechanisms are triggered, including secretion of AMPs, phagocytosis by neutrophils and macrophages and secretion of cytokines and chemokines from numerous cell types, which will orchestrate additional innate and adaptive immune responses.

Innate immune responses are triggered by the detection of microbial structures through pattern-recognition receptors (PRRs) on immune and tissue cells. The most studied PRRs are toll-like receptors (TLRs), which recognize a broad range of bacterial derived macromolecules (Akira and Hemmi, 2003). *S. epidermidis* triggers immune responses partly via TLR-2 (which often forms heterodimers with TLR-1 and TLR-6; Fournier, 2012), similar to *S. aureus* (Yoshimura et al., 1999; Morath et al., 2002). TLR-2 can recognize different bacterial cell wall molecules including lipoproteins, LTA and peptidoglycan (PDG) (Figure 3; Akira et al., 2006; Fournier, 2012), although some of its ligands are still controversial (van Bergenhenegouwen et al., 2013). Secreted components can also be recognized and activate the immune system, as it was shown for *S. epidermidis* PSM, which is recognized by TLR-2/TLR-6 heterodimers (Hajjar et al., 2001).

Recognition of *S. epidermidis* via TLR-2 has been shown in keratinocytes (Wanke et al., 2011; Ommori et al., 2013), endothelial cells (Robertson et al., 2010), or human fibroblasts (Hatakeyama et al., 2003), and has also been demonstrated in TLR-2 transfected human embryonic kidney (HEK)293 cell line (Strunk et al., 2010). Furthermore, in preclinical models of *S. epidermidis* bacteremia or subcutaneous/soft tissue foreign-body infection, an up-regulation of TLR-2 and the adaptor molecule MyD88 has been observed upon infection (Kronforst et al., 2012; Svensson et al., 2015, 2017). The use of TLR-2 knock-out (KO) in bacteremia models with neonatal and adult mice resulted in delayed clearance, especially at early time-points after infection (Strunk et al., 2010; Bi et al., 2015; Cole et al., 2016). These data suggest that TLR-2 is involved in the early responses to *S. epidermidis* infections although is not essential for clearance of the infection (Cole et al., 2016).

Responses toward *S. epidermidis* can also occur independently of TLR-2, as it was shown in the models using TLR-2 KO mice (Bi et al., 2015). Other PRRs that may potentially be involved in *S. epidermidis* sensing are NOD-like receptors, as they recognize *S. epidermidis*-derived PDG (Natsuka et al., 2008). CD14, expressed mostly in monocytes and macrophages, is a TLR-2 co-receptor which may contribute to *S. epidermidis* recognition in some cell subsets (Hatakeyama et al., 2003). PSMs produced by *S. epidermidis* can be sensed by formyl peptide receptor 2 (FPR2/ALX) (Kretschmer et al., 2012, 2015), expressed in neutrophils and involved in their recruitment to the infection site (Rautenberg et al., 2011). To date, the contribution of these receptors *in vivo* has not been addressed.

#### Induction of antimicrobial peptides (AMPs)

Human AMPs are a heterogeneous group of amphipathic peptides, which may be subdivided depending on their structure and function. AMPs functions include rapid, direct killing of microbes and activation/modulation of immune responses, such as cell recruitment or chemokine production. One of the most effective early responses of the host to pathogenic insults is mediated through human β-defensins (hBD). *In vitro* experiments with keratinocytes or skin explants have shown that *S. epidermidis* or its culture supernatants can elicit high levels of hBD-2 and hBD-3 but not hBD-1 (Lai et al., 2010; Li et al., 2013; Ommori et al., 2013; Percoco et al., 2013; Park et al., 2014), and RNase7 and cathelicidin LL-37 in epithelial cells (Burgey et al., 2016). This AMP induction may be beneficial under healthy conditions to counteract more pathogenic species (Lai et al., 2010; Li et al., 2013) but can be also expected to contribute to defense in *S. epidermidis* superficial or ocular infections. Of relevance, some of them (hBD-2, hBD-3, LL-37 and human alpha defensin (HNP)-1) have been proven, to different extents, to be effective against *S. epidermidis in vitro* (Turner et al., 1998; Gordon et al., 2005; Huang et al., 2007; Dapunt et al., 2016b), although no data is available from *in vivo* studies. Nevertheless, the studies mentioned above showed some discrepancies in terms of AMP killing capacity, which could be explained by differences in strains used, as some of them may possess mechanisms against AMP. More relevant in the context of *S. epidermidis* DRI, other cell types including neutrophils and monocytes can produce AMPs. These AMPs will often be located in the phagolysosomes, where they can contribute to bacteria killing. Of interest, hBD-3, LL-37 and hepcidin 20, a liver-derived AMP, have been shown to reduce *S. epidermidis* attachment and/or biofilm formation *in vitro* (Hell et al., 2010; Zhu et al., 2013; Brancatisano et al., 2014). The mechanisms of action is currently unknown, although for hBD-3 a decrease in icaA and icaD expression and increase of icaR were associated with the observations (Zhu et al., 2013).

#### Phagocytosis/killing by neutrophils and macrophages

Phagocytosis by neutrophils is one of the most important mechanisms for elimination of contaminating or infecting bacteria. Neutrophils migrate to the site of infection, following host signals (e.g., chemokines, AMPs) or sensing bacterial components as mentioned above. At the infection site, neutrophils will internalize opsonized bacteria forming a phagosome and, finally, bacteria will be destroyed in the phagolysosome by the action of reactive oxygen species (ROS), proteases and AMPs. An additional mechanism to kill bacteria has been described for neutrophils: the generation of neutrophil extracellular traps (NETs) or NETosis. Nuclear and mitochondrial DNA is released to the extracellular space to form NETs, which contain high local concentrations of intracellular antimicrobial proteins. Although literature is still limited, *S. epidermidis* biofilms have been shown to induce DNA release and NETosis *in vitro* (Meyle et al., 2012; Dapunt et al., 2016a). Macrophages are also able to phagocytose and destroy *S. epidermidis* (Riool et al., 2014) with similar mechanisms, and further present antigens to T cells. Phagocytosis of *S. epidermidis* by macrophages is enhanced following stimulation with IFN-γ *in vitro* (Magrys et al., 2015) and *in vivo* (Boelens et al., 2000a).

Phagocytes will also act against biofilms. It has been shown that neutrophils can bind to opsonized but also non-opsonized biofilms, partly by recognizing EPS (Meyle et al., 2012). Nevertheless, it is generally accepted that the biofilm mode of growth will protect bacteria from phagocytosis, despite some discrepancies in the literature that have been discussed elsewhere (Nguyen et al., 2017). Furthermore, biofilm mode of growth, most often studied in PIA-producing strains, has been shown to decrease killing efficiency in macrophages and neutrophils (Vuong et al., 2004c; Cerca et al., 2006; Kristian et al., 2008; Spiliopoulou et al., 2012).

Interesting observations were made when comparing the phagocytosis of *S. epidermidis* and *S. aureus* biofilms, with the latter being more likely infiltrated and engulfed (Guenther et al., 2009). However, although *S. aureus* was more likely phagocytosed, this does not always correlate with the capacity of neutrophils to kill the bacteria. In fact *S. aureus* has several mechanisms to avoid lysis by neutrophils and to persist intracellularly (Foster, 2005). *S. epidermidis* does not appear to possess similar mechanisms. However, some strains are killed less efficiently, potentially by having a low capacity to prime the oxidative response of neutrophils (Nilsdotter-Augustinsson et al., 2004), or as described before by their biofilm mode of growth. These observations, together with lower induction of neutrophil apoptosis, may lead to intracellular survival and could partially explain the low inflammatory nature and chronicity often associated with *S. epidermidis* infections.

#### Cytokine and chemokine secretion

Cytokines are a broad group of secreted proteins that play a role in intercellular communication, with a broad range of functions within the immune system as cell recruitment, differentiation and activation. Interleukins and other factors play an essential role in leukocyte communication and differentiation, while chemokines are mainly involved in cell recruitment. *In vitro* stimulation of peripheral blood mononuclear cells with different staphylococcal species showed a rapid release of pro-inflammatory cytokines such as IL-1β, IL-6, IL-12p70, or IFN-α (Megyeri et al., 2002). Of note, *S. epidermidis* induced lower levels of pro-inflammatory cytokines compared to *S. aureus* (Megyeri et al., 2002). Studies with monocytes/macrophages have also observed IL-6, tumor necrosis factor (TNF)-α and IL-1β release after *S. epidermidis* stimulation (Wilsson et al., 2008; Strunk et al., 2012). Laborel-Préneron et al. reported that stimulation of moDC with commensal *S. epidermidis* induced a more anti-inflammatory profile in contrast to stimulation with commensal strains of *S. aureus*, with high levels of IL-10 being a key differentiator. Nevertheless, pro-inflammatory cytokines such as IL-6 and TNF-α were also detected (Laborel-Preneron et al., 2015). Similar observations have been made from *in vivo* studies: IL-6, TNF-α, and IL-1β are typically observed in serum in the first hours post-challenge with live or inactivated *S. epidermidis* (Wakabayashi et al., 1991; Simojoki et al., 2011; Bi et al., 2015; Ferreirinha et al., 2016; Qin et al., 2017), or in tissue exudates/homogenates from experimental DRI models (Boelens et al., 2000b; Svensson et al., 2015). The regulatory cytokine IL-10 is also present *in vivo* (Ferreirinha et al., 2016) and it has been shown that *S. epidermidis* inoculation result in higher IL-10 levels compared to *P. aeruginosa* in an intradermal infection model (Bialecka et al., 2005). In a *S. epidermidis* DRI mouse model it was shown that IL-10 was involved in reducing infection-associated morbidity, with higher levels of pro-inflammatory cytokines and greater weight loss in IL-10 KO animals. Interestingly, bacterial counts were the same in both wild-type and KO strains, suggesting that IL-10 does not impact bacterial clearance (Gutierrez-Murgas et al., 2016). Overall, despite differences due to different *S. epidermidis* strains and its effect in different tissues, it can be hypothesized that lower induction of pro-inflammatory cytokines together with high IL-10 production, can contribute to the sub-acute nature of *S. epidermidis* infections.

Multiple chemokines are also released upon *S. epidermidis* infection. Secretion of IL-8, important for neutrophil recruitment, has been described *in vitro* and in the first hours post-infection in *in vivo* studies (Wakabayashi et al., 1991; Boelens et al., 2000b; Simojoki et al., 2011; Svensson et al., 2015). CXCL-1 and CXCL-2, mostly produced by macrophages (via TLR-2 recognition but also by other mechanisms), have also been observed in bacteraemia and peritonitis models (Strunk et al., 2010; Bi et al., 2015; Ferreirinha et al., 2016; Qin et al., 2017). Additionally, a murine peritonitis model revealed increasing levels of numerous chemokines upon challenge with *S. epidermidis* supernatants (Perks et al., 2016).

#### Platelet activation/aggregation

The aggregation and activation of platelets in the presence of bacteria was first described over 25 years ago (Usui et al., 1991) and yet the nature of this interaction has only recently been elucidated. Platelets and bacteria can interact in three ways: the indirect binding of bacteria to a plasma protein (which is a ligand of a platelet receptor), the direct recognition of bacteria by platelet receptors and the binding of secreted bacterial products to platelets (Hamzeh-Cognasse et al., 2015). Only the first type has been described for *S. epidermidis*, where the SdrG has been described to bind platelets in a fibrinogen and Ig-dependent manner; an interaction that leads to platelet aggregation (Brennan et al., 2009). *S. aureus* or *Streptococcus* have been shown to interact with platelets in other ways, which can lead to sepsis or thrombosis but also can play a role in internalization of bacteria by platelets or release of antimicrobial components and immunomodulatory factors (Hamzeh-Cognasse et al., 2015). Future studies will be required to elucidate if *S. epidermidis*-platelets interaction is limited to SdrG or if, like other bacteria, possess multiple mechanisms.

### Adaptive immune response during infection

Adaptive immunity refers to antigen-specific and long-lasting immune responses that are mediated by lymphocytes. Adaptive immunity can be broadly divided in cellular responses, represented by T helper (Th) and cytotoxic T lymphocytes, and humoral responses, represented by B lymphocytes and antibodies. Classically, extracellular bacterial infections have been shown to trigger mostly Th1 cell responses, but more recently Th17 responses have also been linked to the clearance of bacterial infections. Of relevance, an *in vivo* model using immunocompromised mice have shown a higher susceptibility for *S. epidermidis* DRI in mice lacking T cells or T and B cells (Vuong et al., 2008), highlighting a role for adaptive immune responses in infection clearance.

Arising from its status as a commensal microorganism, *S. epidermidis* is expected to elicit adaptive immune responses in humans from early in life. This has been proposed to be largely triggered by a pattern of transient self-resolving infections due to micro-invasions, rather than resulting from local response due to colonization (Brown et al., 2014), but the latter cannot be excluded. These life-long interactions will lead to the generation of an antibody repertoire and a set of memory T and B cells that may confer partial protection from infection. Generation of adaptive immune responses require the presentation of antigens to T cells by antigen presenting cells (APCs), primarily dendritic cells (DC), which will also contribute to T cell polarization. It has previously been shown that CD103+ skin-resident DC, upon interaction with commensal *S. epidermidis*, generates CD8+IL-17A+ T cells with the capacity to enhance protective responses in the skin (Naik et al., 2015). Upon infection, it can also be expected that certain DC subtypes, already present in the tissue or that will migrate there, will shape adaptive immune responses. Data available for *S. epidermidis* interaction with DC is very limited but it has been observed, *in vitro* and *in vivo*, that *S. epidermidis* can lead to DC activation with an increase in co-stimulatory molecules such as CD86 or CD80 and antigen presenting molecules such as major histocompatibility complex (MHC)-II (Stanislawska et al., 2005; Cerca et al., 2014; Laborel-Preneron et al., 2015; Franca et al., 2016). Studies describing cytokine secretion by DC stimulated with *S. epidermidis* (whole bacteria or its secreted proteins) have yielded somewhat inconsistent results. For example, IL-10 was not highly secreted when bone-marrow DC were stimulated with *S. epidermidis* (Cerca et al., 2014), but the stimulation of moDC with *S. epidermidis* secreted proteins led to high IL-10 secretion (Laborel-Preneron et al., 2015). The inconsistency between these reports may be due to the different sources of DC and stimuli used, which can lead to different outcomes by activating distinct pathways. The relevance of the stimuli is further highlighted in a series of experiments from Durantez et al. *S. epidermidis* PSM-derived peptides combined with ovalbumin were able to trigger cytotoxic T cell responses, however, this was only observed after those peptides were presented via APCs together with stimuli specific for TLR-3, TLR-7, and TLR-9 (Durantez et al., 2010). Further experiments are required to clarify the exact role of APCs and different DC subsets in priming and polarizing the T cell response.

With regards to humoral responses, antibodies against *S. epidermidis* proteins have been detected in serum and saliva of healthy individuals (Sadovskaya et al., 2007; Carvalhais et al., 2015), but levels are generally lower compared to *S. epidermidis* infected patients (Sadovskaya et al., 2007). Antibodies against biofilm components and cytoplasmic proteins have been found to be predominant (Carvalhais et al., 2015).

To assess the potential use of antibody titers in diagnosis of infection, serum antibody titters against Staphylococcal proteins have been measured in patients with *S. aureus* or *S. epidermidis* infections (such as wound infections, bacteremia or DRI). Recently, a multiplex antibody detection-based immunoassay was evaluated for the diagnosis of peri prosthetic joint infections (PJI). The assay included protein antigens from several strains: diverse Staphylococci, *Streptococcus agalactiae* and *P. acnes* (Marmor et al., 2016). The test showed a slightly lower sensitivity than C-reactive protein and erythrocyte sedimentation rate, however was able to diagnose around 50% of patients, which were culture positive but presented low systemic inflammation values (Marmor et al., 2016).

Another goal of humoral response studies is to identify immunogenic proteins, which can lead to development of therapeutic and/or prophylactic treatments. Studies employing 2D protein electrophoresis or phage display technology with the aim of identifying *S. epidermidis* immunogenic proteins have been performed in rabbits (Sellman et al., 2005) and humans (Pourmand et al., 2006). Sera of rabbits immunized with live *S. epidermidis* were used to detect relevant immunogens. Mice were then immunized with several selected proteins, five of whom (Na+/H+ antiporter, Acetyl-CoA C-acetyltransferase, lipoate ligase, cysteine synthase and alanine dehydrogenase) lead to a significant reduction of bacterial loads in a murine infection model (Sellman et al., 2005). Other proposed immunogenic proteins include AtlE, Staphylococcal conserved antigen B (ScaB), and GehD lipase, which elicited higher antibody titers in infected patients compared to non-infected subjects. Active immunization of mice with these antigens resulted in production of specific antibodies with *in vitro* opsonization capacity against *S. epidermidis* (Pourmand et al., 2006). An anti-SdrG antibody was shown to reduce mortality in a neonate bacteremia rat model and to decrease bacterial counts in a DRI (endocarditis) rabbit model (Vernachio et al., 2006), although it failed in a clinical trial to prevent late-onset sepsis in low-birth weight neonates (Schaffer and Lee, 2009). More recently it was shown that immunization with staphylococcal Major amidase (Atl-AM), a cell wall hydrolase present in some *S. epidermidis* and *S. aureus* strains, increases antibody levels against that protein in mice (Nair et al., 2015). In the same study, immunized animals challenged with a lethal intraperitoneal dose of *S. epidermidis* showed a better survival and lower bacterial counts in tissues compared to mock immunized animals (Nair et al., 2015). Additionally, immunized mice also presented higher levels of Th1 and Th2 cells, although it did not elucidate which responses were the most relevant for the increased survival. Immunizations with Aap or with antibodies against surface proteins have also been shown to reduce colonization in a murine DRI model by ultimately inhibiting biofilm formation (Shahrooei et al., 2012; Yan et al., 2014). Despite the fact that their efficacy against *S. epidermidis* infections has not been tested *in vivo*, antibodies against PNAG/PIA and phosphonate ABC transporter substrate binding protein (PhnD) have shown efficacy against *S. epidermidis* biofilm formation *in vitro* (Franca et al., 2013; Lam et al., 2014). A recent study focused on staphylococcal adhesion proteins, which contain long stretches of Sdr and are key virulence factors for *S. epidermidis* and also *S. aureus*. The study led to the discovery of two novel bacterial glycosyltransferases, SdgA and SdgB, which can modify all Sdr-proteins to protect them from cleavage by cathepsin G (a neutrophil protein). Neutralization of these enzymes may be the next opportunity for an effective anti-staphylococcal approach (Hazenbos et al., 2013). To date, all anti-staphylococcal antibodies tested against *S. epidermidis* and other CoNS in clinical trials (Altastaph, INH A-2, and Pagibaximab) have been found to be ineffective in reducing bacteremia in neonates (Patel and Kaufman, 2015). Although there is still much work to be done to fully understand effective immune responses against *S. epidermidis*, on-going research offers several candidates and strategies to develop new therapeutic products.

Additionally, there are also T cell-mediated immune responses to *S. epidermidis* although they are poorly characterized. Based on *in vitro* studies, it has been suggested that *S. epidermidis* opsonization with IgG promotes Th17 responses (den Dunnen et al., 2012), although the role of this phenomenon *in vivo* has not been shown. On the other hand, in an *in vivo* model of foreign-body infection, a beneficial effect of IFN-γ injections has been shown, suggesting a protective role of Th1 dominated responses in bacterial infections (Boelens et al., 2000a). Based on cytokines induced by *S. epidermidis* in the different studies (e.g., IL-6, IFN-γ, or IL-12), a Th1/Th17 polarization may be expected in such infections. This goes in line with the findings of Ferreirinha *et al*., who observed that injection of PNAG-producing *S. epidermidis* in mice lead to IFN-γ and IL-17A producing T cells (Ferreirinha et al., 2016). Also, as mentioned above, immunization of mice with Atl-AM led to an increase in Th1 and Th2 cells (Th17 cells were not evaluated on that study). Immunization also led to a higher survival; however, direct effect of T cell responses in that finding was not further addressed (Nair et al., 2015).

### Bone system interactions

**Figure 4:**
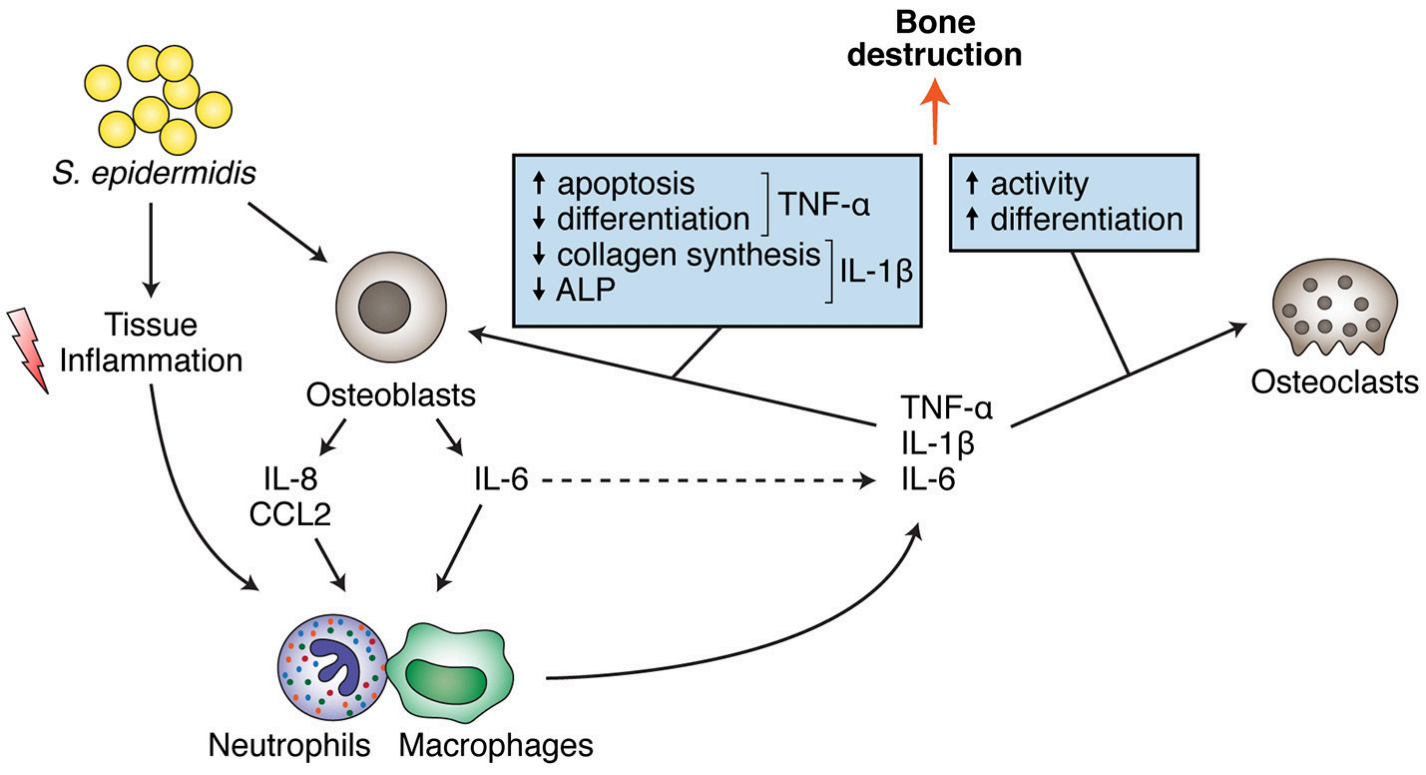
*S. epidermidis* direct and indirect effects on bone cells (osteoblasts and osteoclasts), leading to bone destruction.

The usual chronic nature of *S. epidermidis* osteomyelitis will eventually lead to an inflammatory environment within the bone system, which is of special relevance in the context of ODRIs. Bone as an organ is particularly sensitive to chronic inflammation, due to its continuous remodeling process that is influenced by different components of the immune system and inflammatory pathways (Redlich and Smolen, 2012). Due to their potent capacity to stimulate the formation and activity of bone resorbing osteoclasts, pro-inflammatory cytokines such as TNF-α, IL-1β, and IL-6 (Raisz, 1999; Kobayashi et al., 2000; Lam et al., 2000) are powerful drivers of osteolysis. Conversely, the function of the bone matrix-producing cells, osteoblasts, is also negatively affected by pro-inflammatory cytokines, such as TNF-α (Jilka et al., 1998; Gilbert et al., 2000, 2002) or IL-1β (Stashenko et al., 1987; Figure 4). Therefore, persistently elevated levels of pro-inflammatory cytokines in the local bone microenvironment frequently result in marked osteolysis, driven by enhanced osteoclast activity at the site of infection (Figure 4; Nair et al., 1996), which is likely compounded by a diminished capacity of osteoblasts to produce new bone matrix.

Despite the importance of *S. epidermidis* as a causative agent in ODRI, relatively little information exists about the interactions of *S. epidermidis* with resident bone cells, in particular the molecular mechanisms underlying the bone loss observed in *S. epidermidis*-induced osteomyelitis. The production of cytokines by innate and/or adaptive immune cells in response to *S. epidermidis* is undoubtedly an important contributor to the enhanced bone resorption observed at the site of infection, however it is becoming apparent that the osteoblast itself may also directly contribute to the production of pro-inflammatory cytokines and therefore further perturb the balance of bone formation and resorption in favor of bone destruction. A recent study has shown the induction *in vitro* of IL-6 by primary human osteoblasts stimulated with *S. epidermidis* (Dapunt et al., 2016b). *S. epidermidis* infection also induced chemokines, such as IL-8/CXCL8 and CCL2/MCP-1, suggesting that osteoblasts may be capable of further recruiting immune cells following an encounter with *S. epidermidis*. Interestingly, the authors also demonstrated that osteoblasts were activated not only by the planktonic form of *S. epidermidis* but also by components of *S. epidermidis* biofilms. This suggests that, rather than the relatively simplistic view of the osteoblast for producing bone matrix and regulating osteoclast activity, osteoblasts may also serve an important role as sensors and initiators of immune responses directed against bacteria resident in the local bone microenvironment.

Additionally, *in vitro* studies have observed a decrease in osteoblast viability when co-cultured with *S. epidermidis* (Lee et al., 2010; Zaatreh et al., 2016). *S. epidermidis* products (resulting from washing bacteria) have been suggested to induce bone destruction as they increased calcium release from murine bones *in vitro* (Meghji et al., 1997). This is in stark contrast to *S. aureus*, which has been extensively studied in this context and is capable of influencing the behavior of both osteoblasts and osteoclasts. For example, *S. aureus* has been demonstrated to induce TRAIL-dependent apoptosis in osteoblasts (Tucker et al., 2000; Alexander et al., 2001, 2003; Young et al., 2011) and can stimulate expression of osteolytic factors (Somayaji et al., 2008) or reduce the expression of its inhibitors (Young et al., 2011), exacerbating the osteolytic effect. Furthermore, specific bacterial proteins have been identified as responsible for some of these effects on osteoblasts such as *S. aureus* protein A, which has been demonstrated to bind directly to TNF receptor 1, resulting in an inhibitory effect on proliferation, the induction of apoptosis, and the stimulation of RANKL expression (Claro et al., 2011, 2013).

Regarding the effects of bacterial infection on osteoclasts, a number of studies have reported the effects of inactivated *S. aureus*, or specific *S. aureus* components, for affecting osteoclast formation and/or activity (Yang et al., 2009; Pietrocola et al., 2011; Kishimoto et al., 2012; Kim et al., 2013). Conversely, staphylococcal LTA inhibits osteoclastogenesis through stimulation of TLR-2 activity (Yang et al., 2009). Such conflicting data strongly argues for the use of (preferably live) intact bacteria in such osteoclastogenesis assays rather than purified bacterial components. When the effect of intact bacteria on osteoclastogenesis was recently investigated, *S. aureus* was demonstrated to have both direct and indirect stimulatory effects on osteoclasts *in vitro* (Trouillet-Assant et al., 2015). By inducing activation of macrophages and thereby stimulating the production of pro-inflammatory cytokines, *S. aureus* indirectly enhanced the formation of osteoclasts from precursor cells. Additionally, *S. aureus* could also directly infect mature osteoclasts, resulting in increased cell fusion and enhanced bone resorbing capacity. Much less is known regarding direct interaction of *S. epidermidis* and osteoclasts, although it is expected that induction of pro-inflammatory cytokines will enhance bone destruction in similar ways.

Given the multitude of different effects of *S. aureus* on osteoblast and osteoclast function, it is likely that *S. epidermidis* can also negatively affect the capacity of osteoblasts to produce bone matrix and/or enhance osteoclast formation and function, although much further work is necessary to clarify if this is indeed the case.

Lastly, the interaction of *S. epidermidis* with bone cells could provide a location where bacteria can persist and prolong ODRIs. Both *S. aureus* and *S. epidermidis* are capable of invading osteoblasts *in vitro* (Ahmed et al., 2001; Khalil et al., 2007), however the mechanism underlying this phenomenon appears to differ between these two species. *S. aureus* requires binding to the ECM protein fibronectin, mediated by α_5_β_1_ integrin (Sinha et al., 1999), whereas *S. epidermidis* internalization by osteoblasts is not affected by interfering with fibronectin binding or blocking, suggesting a different mechanism (Khalil et al., 2007). This is supported by the findings of a recent study that reported SdrG mediates the binding of *S. epidermidis* to osteoblasts *in vitro*, an effect likely mediated through SdrG binding to α_V_β_3_ integrin (Claro et al., 2015). However, this immune evasion mechanism may be of more importance for *S. aureus* rather than *S. epidermidis per se*, since the capacity of *S. epidermidis* for invading osteoblasts *in vitro* does not appear to differ between commensal strains and clinical isolates of *S. epidermidis* obtained from infected orthopedic devices (Valour et al., 2013). This is reinforced by a recent *in vitro* study demonstrating that *S. epidermidis* as well as other opportunistic pathogens such as *S. lugdunensis* and *Enterococcus faecalis* were incompetent at being internalized by MG63 human osteoblastic cells, being internalized at a level approximately three orders of magnitude lower than that observed with *S. aureus* (Campoccia et al., 2015). Osteoclasts are also able to internalize, at least, *S. aureus*. Given the inherent phagocytic capacity of osteoclasts, it may be that internalization of *S. aureus* by osteoclasts relies on such a phagocytic mechanism of uptake. This raises the possibility that *S. epidermidis* may also be the object of uptake by osteoclasts. Together with the previously stated ability of *S. epidermidis* to bind to α_V_β_3_ integrin, which is highly expressed by osteoclasts (Quinn et al., 1991), this further suggests that *S. epidermidis* may bind to and be internalized by osteoclasts, although this and the subsequent phenotypical changes resulting from such an interaction requires to be validated experimentally. Taken together, this suggests that while the persistence of orthopedic implant-associated *S. aureus* infections *in vivo* may well stem from its enhanced ability to invade osteoblasts, and potentially osteoclasts, other mechanisms, such as biofilm formation, may underlie the persistence of *S. epidermidis* in implant-related infection.

Finally, the integration of immune responses within the bone system in the context of *S. epidermidis* infection has been largely unexplored. The number of models described for *S. epidermidis* bone infection is limited (Table 1) and none have really focused on host immune responses. Most of the data available is based on *S. aureus* models, where a combination of Th1/Th17 responses have been observed (Prabhakara et al., 2011a; Rochford et al., 2016), although it is not clear if this response is beneficial or detrimental to the host as no bacterial clearance was achieved (Prabhakara et al., 2011b; Jensen et al., 2015). The observation that anti-IL-12p40 conferred protection in *S. aureus* infected C57BL/6 mice supported the hypothesis that skewed Th1/Th17 responses may be harmful (Prabhakara et al., 2011b), as IL-12/IL-23p40 plays a role in polarization of these cell types. This observation, however, could be due to a decrease in myeloid-derived suppressor cells (MDSC) that otherwise would impair immune responses in the vicinity of an implant, as described by Heim et al. (2015). The use of different murine strains, inoculum dose and models are factors contributing to the disparity in the available data. Furthermore, the differences between *S. aureus* and *S. epidermidis* are quite significant, and so further work focused on *S. epidermidis* is required to provide a proper understanding of adaptive immune responses to *S. epidermidis* bone infection.

**Table 1:** Bone-related infection models with *S. epidermidis* as infective agent.

**Species**	**Gender, Age/weight**	**Strain**	**CFU dose**	**Inoculation method**	**Study purpose**	**Findings**	**Comments**	**References**
Mouse	ND	ND	10^8^ CFU	Bacteria inoculated at the end of the wire (joint area)	Protocol available only	NA	PJI model	Scherr et al., 2014
Wistar rat	Male 250 – 300 g	IDRL-8883 clinical isolate (MRSE strain)	10^7^ CFU and a colonized wire	Bacteria injected (0.1 ml) into the tibia and a pre-colonized wire was implanted	Establish a model of foreign body-associated osteomyelitis to test Tedizolid treatment and to compare with standard treatment	Tedizolid alone presented better results than vancomycin monotherapy. Addition of rifampin to both treatments increased effectivity of therapy	No fracture Addition of sclerosing agent	Park et al., 2016
Wistar rat	Male 12-week old	Clinical isolate (MRSE strain)	10^3^, 10^5^, and 10^8^ CFU	Bacteria injected (0.03 ml) into femoral defect	Establish a model to study *S. epidermidis* non-unions	Low-grade *S. epidermidis* contamination can prevent bone healing, even in the absence of infectious signs	Bone osteotomy performed Self-clearance in some animals from low dose group (33%)	Lovati et al., 2016b
Wistar rat	Male 12-week old	Clinical isolate (MRSE strain)	10^5^ CFU	Bacteria injected (0.03 ml) into femoral defect	Test systemic and local administration of vancomycin or mesenchymal stem cells on infection		Bone osteotomy performed	Lovati et al., 2016a
Wistar rat	ND 350 – 450 g	*S. epidermidis* ATCC 35984	10^4^ CFU	Bacteria injected into surgical site before wound closure (calvarial defect reconstituted with different materials)	Compare silicon nitride implants with titanium and PEEK implants in terms of bone formation and prevention of infection	Silicon nitride implants showed higher osteointegration and lower presence of live bacteria	Only histological findings with a very small size group	Webster et al., 2012
Sprague-Dawley rat	Male, adult 425 ± 37 g	Clinical isolates of *S. epidermidis* and *S. aureus*	1.5 × 10^7^ (*S.epidermidis*) 1.5 × 10^4^ (*S.aureus*)	Bacteria injected (0.05 ml) through a PTFE catheter into tibia medullary canal Catheter left on place	To test ^68^Ga-DOTA-Siglec-9 PET/CT imaging in *S. aureus* and *S. epidermidis* infection	^68^Ga-DOTA-Siglec-9 PET/CT was able to detect tissue inflammation but not able to distinguish *S.aureus* from *S.epidermidis* infections	No fracture 5% sodium morrhuate added before inoculation in *S. epidermidis* group but not in *S. aureus* or control group	Ahtinen et al., 2014
New Zealand White rabbit	ND 2.5 – 3.5 kg	*S. epidermidis* ATCC 35984	10^3^, 10^4^ and 10^5^ CFU (pilot study) 10^4^ CFU (main study)	Bacteria injected (in 1 ml) into knee joint, near inserted implants (stainless-steel screw and UHMWPE washer)	To study the effect of Allicin (antibacterial principle of garlic) in biofilm formation in a prosthetic joint infection model	Allicin alone and in combination with Vancomycin were effective in reducing biofilm formation	PJI model	Zhai et al., 2014
New Zealand White rabbit	Female, adult 2.46 ± 0.23 kg	Clinical isolate (MRSE strain)	10^7^ CFU	Bacteria injected (in 0.1 ml of saline) into tibia medullary cavity Afterwards, a bone cement cylinder was inserted	To test effectivity of chitosan loaded PMMA bone cements *in vivo*	Quaternized chitosan-loaded PMMA was able to reduce scoring and CFU counts when compared to sole, gentamicin or chitosan loaded PMMAs	No fracture	Tan et al., 2014
New Zealand White rabbit	Male, skeletally mature 3.2 ± 0.37 kg	Clinical isolates and *S. epidermidis* ATCC 35983	10^8^ CFU (*S.epidermidis*) 10^4^ CFU (*S.aureus*)	Bacteria injected (in 0.1 ml) into tibia medullary space next to a cement block	To test ^18^F-FDG PET/CT imaging in *S.aureus* and *S.epidermidis* infection	*S. epidermidis* infection presented low ^18^F-FDG uptake due to limited leukocyte infiltration	No fracture 5% sodium morrhuate added in medullary canal in *S.epidermidis* groups only	Lankinen et al., 2012
New Zealand White rabbit	Male 2.5 – 3.5 kg	*S. epidermidis* Xen43, bioluminescent strain derived from a clinical isolate	10^4^ CFU	Bacteria injected (in 0.1 ml of saline) into tibia medullary cavity where an intramedullary electrode was placed	To compare the electricidal effect with an antibiotic treatment	Electrical current was as effective as intravenous doxycycline treatment in a foreign-body infection model	No fracture	Del Pozo et al., 2009
New Zealand White rabbit	Male 4.0 ± 0.5 kg	*S. epidermidis* RP-62A Clinical isolate?	–	Commercially-pure titanium implants were exposed to a 10^6^ CFU/ml solution for 1 h at 37°C. Implant placed into the lateral femoral condyle	To study the effectivity of cross-linked albumin coating in infection prevention	The albumin coated implants presented a lower infection rate	No fracture Not so clear results: animals where bacteria were detected with gram stain counted as not infected	An et al., 1997
New Zealand White rabbit	Female, adult 3.5 – 4 kg	Clinical isolate of *S. epidermidis*	5 × 10^7^ CFU	Bacteria were injected into femoral medullary canal and drill whole was closed with a stainless steel screw	To test vancomycin and minocycline alone or in combination with rifampin in an orthopedic device related infection model	Vancomycin plus rifampin was the most effective treatment, followed by minocycline plus rifampin. No clearance or very low was used with antibiotics alone	No fracture	Isiklar et al., 1996
New Zealand White rabbit	Male, ND	*S. epidermidis* G109-83 and *Bacteroides thetaiotaomicron* N54-83 and clinical isolate N1660-75B	10^7^ CFU of each strain	Barium-impregnated silicone rubber catheter was introduced into medullary canal and bacteria were injected into it (0.1 ml for each) together or separately. Second group was injected directly into medullary canal without a foreign body	Observe influence of foreign-body in a model of osteomyelitis with *S. epidermis* and *B. thetaiotaomicron*, alone or in combination	Both strains, alone or in combination were able to cause osteomyelitis, however in the presence of a foreign-body the severity of osteomyelitis was higher	No fracture 5% sodium morrhuate added	Mayberry-Carson et al., 1992
New Zealand White rabbit	Male, ND	*S. epidermidis* G109-83 and clinical isolate *Bacteroides fragilis* N17-85	10^7^ CFU of each strain (alone or combined)	Barium-impregnated silicone rubber catheter was introduced into medullary canal and bacteria were injected into it (0.1 ml for each) together or separately	Establish an foreign-body-associated osteomyelitis model with *B. fragilis, S.epidermidis* or combination of both	Both strains, alone or in combination were able to cause osteomyelitis, however *S. epidermidis* appeared less pathogenic than *B. fragilis*	No fracture 5% sodium morrhuate added	Lambe et al., 1991
New Zealand White rabbit	Male, ND	*S. epidermidis* G109-83 and *Bacteroides thetaiotaomicron* N54-83	10^7^ CFU of each strain	Barium-impregnated silicone rubber catheter was introduced into medullary canal and bacteria were injected into it (0.1 ml for each)	Study ciprofloxacin efficacy in polymicrobial osteomyelitis	Ciprofloxacin showed little efficacy in a polymicrobial device-related osteomyelitis	No fracture 5% sodium morrhuate added	Mayberry-Carson et al., 1990
Dog	ND 10 – 5 kg	*S. epidermidis, S. aureus* and *E.coli*	10^2^–10^8^ CFU?	Bacterial suspension was introduced into femoral canal, with or without implants	Study influence of different implants on infection incidence (materials tested)	All materials increased likelihood of *S. aureus* infection, and only PMMA polymerized *in vivo* increased *S. epidermidis* and *E. coli* infection risk.	No fracture	Petty et al., 1985
Goat	ND	Clinical isolate *S. epidermidis*	3 × 10^5^ CFU	Bacterial suspension inoculated (0.1 ml) into wounds around pins placed on tibia	To study the effectivity of electrical current on stainless steel fixator in preventing infection	Small current applied to external fixators decreased the infection percentage	No fracture	van der Borden et al., 2007
Ile-de-France sheep	ND 4 – 9 years	Clinical isolate *S. epidermidis*	1–3 × 10^8^ CFU	Bacteria injected (in 1 ml of PBS) into femur medullary canal Afterwards, a stainless steel implant was inserted (uncoated, hydroxyapatite-coated or PMMA cemented)	To study the effect of hydroxyapatite and PMMA implant coatings on infection progression	Higher infection rate in animals with an hydroxyapatite-coated implant	No fracture	Laure et al., 2008

*CFU, Colony Forming Unit; PEEK, poly(ether ether ketone); PTFE, polytetrafluoroethylene; ^68^Ga-DOTA, ^68^Ga-labeled 1,4,7,10-tetraazacyclododecane-1,4,7,10-tetraacetic acid; Siglec-9, Sialic acid-binding immunoglobulin-like lectin 9; 18F-FDG, 2-deoxy-2-[fluorine-18]fluoro-D-glucose; PMMA, polymethylmetacrylate; NA, Not applicable; ND, Not described; UHMWPE, ultra-high molecular weight polyethylene*.

## Summary and outlook

*S. epidermidis* is a commensal microorganism adapted for the colonization of human skin. In healthy individuals, *S. epidermidis* can provide several benefits by competing with pathogenic species or by modulating the immune system. Induction of tolerance has been demonstrated recently in murine models although similar mechanisms remain to be proven in humans. The great advances in “omics” are providing enormous amounts of data about cell/tissue behavior and also about human microbiome (from transcriptome to metabolome). The application and integration of this data for *S. epidermidis* commensalism will provide a much better understanding of the roles of *S. epidermidis* in health and also in certain skin diseases, such as atopic dermatitis or psoriasis.

Upon a transition to a pathogenic interaction with the host, as occurs in DRI, the same mechanisms that allow *S. epidermidis* to reside in human skin and mucosal tissues allow adhesion and biofilm formation upon the implanted device. Adhesion to host proteins and biofilm formation are thought to be the main *S. epidermidis* pathogenic mechanisms. For this reason, the development of antimicrobial surfaces and therapies targeting biofilm are areas which are expected to be in development in the coming years. In the face of high antibiotic resistance, these technologies may need to consider alternative antimicrobial agents.

Finally, there remains a lack of understanding of immune responses to *S. epidermidis* infections. *S. epidermidis* seems to trigger low levels of pro-inflammatory cytokines secretion and high levels of IL-10, which may contribute to the sub-acute nature and persistence of the infection. As yet, adaptive immune responses to the bacterium remain poorly described and are an area which may provide significant new discoveries in the coming years.

## Author contributions

MSB, LGH, KT, BS, MM, and TFM wrote the manuscript and approved its final version. MSB, and LGH were involved in figures preparation. TFM, LO, and RGR corrected and critically evaluated the manuscript.

### Conflict of interest statement

The authors declare that the research was conducted in the absence of any commercial or financial relationships that could be construed as a potential conflict of interest.

## Abbreviations

Aae: Autolysin/adhesion from *S. epidermidis*
Aap: Accumulation associated protein
AMPs: Antimicrobial peptides
APC: Antigen-presenting cell
AtlE: Autolysin
Bhp: Biofilm associated homolog protein
CoNS: Coagulase-negative staphylococcus
CWA: Cell-wall-anchored
DC: Dendritic cells
DRI: Device-related infection
ECM: Extracellular matrix
eDNA: Extracellular DNA
Embp: Extracellular matrix-binding protein
EPS: Extracellular polymeric substances
FPR2: Formyl peptide receptor 2
IFN: Interferon
IL: Interleukin
KO: Knock-out
LTA: Lipoteichoic acid
moDC: Monocyte-derived dendritic cells
MRSE: Methicillin resistant *S. epidermidis*
MSCRAMMs: Microbial surface components recognizing adhesive matrix molecules
ODRIs: Orthopedic device-related infections
PDG: Peptidoglycan
PIA: Polysaccharide intercellular adhesin
PGA: Poly-γ-glutamic acid
PSMs: Phenol-soluble modulins
RANKL: Receptor activator of NFκB ligand
ROS: Reactive oxygen species
Sdr: Serine-aspartate repeat protein
SCC*mec*: Staphylococcal cassette chromosome *mec*
Ses: *S. epidermidis* surface protein
SCV: Small colony variant
SSP: Staphylococcal surface protein
TNF-α: Tumor necrosis factor alpha
Th: T helper
TLR: Toll-like receptor
WTA: Wall teichoic acids.
